# Characterization of TCF21 Downstream Target Regions Identifies a Transcriptional Network Linking Multiple Independent Coronary Artery Disease Loci

**DOI:** 10.1371/journal.pgen.1005202

**Published:** 2015-05-28

**Authors:** Olga Sazonova, Yuqi Zhao, Sylvia Nürnberg, Clint Miller, Milos Pjanic, Victor G. Castano, Juyong B. Kim, Elias L. Salfati, Anshul B. Kundaje, Gill Bejerano, Themistocles Assimes, Xia Yang, Thomas Quertermous

**Affiliations:** 1 Department of Medicine, Division of Cardiovascular Medicine, Stanford University School of Medicine, Stanford, California, United States of America; 2 Cardiovascular Institute, Stanford University School of Medicine, Stanford, California, United States of America; 3 Department of Integrative Biology and Physiology, University of California, Los Angeles, Los Angeles, California, United States of America; 4 Department of Genetics, Stanford University School of Medicine, Stanford, California, United States of America; 5 Department of Computer Science, Stanford University School of Medicine, Stanford, California, United States of America; 6 Department of Pediatrics, Stanford University School of Medicine, Stanford, California, United States of America; 7 Department of Developmental Biology, Stanford University School of Medicine, Stanford, California, United States of America; University of Oxford, UNITED KINGDOM

## Abstract

To functionally link coronary artery disease (CAD) causal genes identified by genome wide association studies (GWAS), and to investigate the cellular and molecular mechanisms of atherosclerosis, we have used chromatin immunoprecipitation sequencing (ChIP-Seq) with the CAD associated transcription factor TCF21 in human coronary artery smooth muscle cells (HCASMC). Analysis of identified TCF21 target genes for enrichment of molecular and cellular annotation terms identified processes relevant to CAD pathophysiology, including “growth factor binding,” “matrix interaction,” and “smooth muscle contraction.” We characterized the canonical binding sequence for TCF21 as CAGCTG, identified AP-1 binding sites in TCF21 peaks, and by conducting ChIP-Seq for JUN and JUND in HCASMC confirmed that there is significant overlap between TCF21 and AP-1 binding loci in this cell type. Expression quantitative trait variation mapped to target genes of TCF21 was significantly enriched among variants with low *P*-values in the GWAS analyses, suggesting a possible functional interaction between TCF21 binding and causal variants in other CAD disease loci. Separate enrichment analyses found over-representation of TCF21 target genes among CAD associated genes, and linkage disequilibrium between TCF21 peak variation and that found in GWAS loci, consistent with the hypothesis that TCF21 may affect disease risk through interaction with other disease associated loci. Interestingly, enrichment for TCF21 target genes was also found among other genome wide association phenotypes, including height and inflammatory bowel disease, suggesting a functional profile important for basic cellular processes in non-vascular tissues. Thus, data and analyses presented here suggest that study of GWAS transcription factors may be a highly useful approach to identifying disease gene interactions and thus pathways that may be relevant to complex disease etiology.

## Author Summary

While coronary artery disease (CAD) is due in part to environmental and metabolic factors, about half of the risk is genetically predetermined. Genome-wide association studies in human populations have identified approximately 150 sites in the genome that appear to be associated with CAD. The mechanisms by which mutations in these regions are responsible for predisposition to CAD remain largely unknown. To begin to explore how disease-specific gene sequences and disease gene function promotes pathology, we have mapped the loci and genes that are downstream of the transcription factor TCF21, which is strongly associated with CAD. By identifying genes that are regulated by TCF21 we have been able to link together multiple other CAD associated genes and begin to identify the critical molecular processes that mediate atherosclerosis in the blood vessel wall and contribute to the genesis of ischemic cardiovascular events.

## Introduction

Recent large-scale GWAS have identified 46 genome-wide significant CAD loci and a further 104 independent variants associated at a 5% false discovery rate (FDR), yet the biological and disease-relevant mechanisms for these associations remain largely unknown [1]. It is estimated that at least two-thirds of the disease loci contain causal genes that are not related to known cardiovascular risk factors such as diabetes and lipid metabolism, suggesting that they are involved in disease promoting processes in the blood vessel wall. Thus the great promise of these genetic findings is the elucidation of atherosclerosis disease pathways, and further investigation of mechanisms by which genes in disease loci work together to regulate cellular and molecular functions that are involved in disease risk is sorely needed. Among the significant loci, a small subset of genes encode transcription factors (TFs) which are likely to impact disease risk by regulating disease relevant genes and possibly other CAD associated genes. Further study of downstream targets of these TFs, employing well established genome-wide methods, would be expected to provide biological insights through links to established pathways and to identify informative relationships among other apparently independent causal CAD loci.

Tcf21 is a member of the basic helix-loop-helix (bHLH) TF family and is critical for the development of a number of cell types during embryogenesis of the heart, lung, kidney, and spleen [2–5]. *Tcf21* is expressed in mesodermal cells in the proepicardial organ that give rise to coronary artery smooth muscle cells (SMC) and loss of *Tcf21* results in increased expression of smooth muscle markers by cells on the heart surface consistent with premature SMC differentiation [6]. Knockout animals also exhibit a dramatic failure of cardiac fibroblast development suggesting a role for *Tcf21* in the fate decisions of a precursor cell for SMC and cardiac fibroblast lineages [2,6]. These data are consistent with the hypothesis that early expression of *Tcf21* is important for expansion of the SMC compartment of the coronary circulation, with persistent *Tcf21* expression being required for cardiac fibroblast development [2,6].

To better understand the cellular functions of TCF21 in the SMC lineage, and to gain insights into how such functions might contribute to CAD risk, we performed chromatin immunoprecipitation coupled with high throughput sequencing (ChIP-Seq), examined the downstream target loci and genes that harbor TCF21 binding sites, and employed bioinformatic and experimental approaches to investigate how the target genes work together to mediate the risk of CAD. Pathway analysis of downstream target genes revealed that TCF21 regulates cell-cell and cell-matrix interactions as well as growth factor signaling pathways. Also, we found TCF21 target regions to be over-represented among CAD associated loci, and that genes in these regions assemble into pathways that mediate fundamental processes such as cell cycle, chromatin remodeling, and growth factor signaling. Taken together, these studies elucidate disease-associated genes and pathways that lie downstream of TCF21, and show how SMC related processes may be responsible for a substantial portion of the genetic risk for CAD.

## Results

### ChIP-Seq studies identify TCF21 core target loci and genes

Our primary interest in these studies was the transcriptional network of TCF21-regulated genes contributing to the development of human CAD. Because of the known role of TCF21 in the embryonic development of coronary vascular SMC, we undertook these ChIP-Seq experiments in primary cultured human coronary artery SMC (HCASMC). Furthermore, we selected culture conditions that maintain these cells in the synthetic, undifferentiated state that most closely reflects the disease phenotype [7]. Two polyclonal antibodies raised against peptides representing different epitopes of TCF21 and previously validated by the manufacturers were employed in these studies. ChIP-Seq was performed with both antibodies (Ab1 and Ab2), with two replicates per antibody and an IgG control condition. We then followed best practices for computational analysis of sequence data as put forth by the ENCODE project, including genome alignment, peak calling, and replicate consolidation using the Irreproducible Discovery Rate (IDR) method to identify high confidence peaks for each antibody [8].

ChIP-Seq using Ab1 identified 10,523 peaks while Ab2 identified 4,900 peaks that largely overlapped with those identified by Ab1. These two sets of peaks were within 50 kb of 12,226 and 7,150 genes, respectively (Table 1). To better understand the disparity between the numbers of DNA regions immunoprecipitated by the two antibodies, we characterized the distribution and relationship between the two sets of peaks identified. The spatial distribution of each peak set was investigated by graphing the distance between peaks and the transcription start site of the nearest gene (S1A and S1B Fig). These distributions were nearly identical for the two peak sets. In each case, peaks were distributed primarily within 100 kb of the transcription start sites, with 90% of peaks being found within this interval. The similarity between antibody peak localization was further demonstrated by relating the peak coordinates to structural gene features (S1C and S1D Fig). The pattern of distribution for peaks associated with both antibodies revealed the majority of binding sites were located within intronic and intergenic regions, with a significant number of peaks also being found within the promoter and exonic regions and a very small number of peaks mapping to transcript untranslated sequences.

**Table 1:** TCF21 binding characteristics with different antibodies alone and when individual antibody data is merged.

Data	Peak counts	Average lengths	Target genes	Genes with >1 peak	Peaks with >1 gene
Ab1	10523	614	12226	5371	5474
Ab2	4900	483	7150	2055	2464
Ab_Shared	4852	463	7128	2042	2457

**Fig 1:**
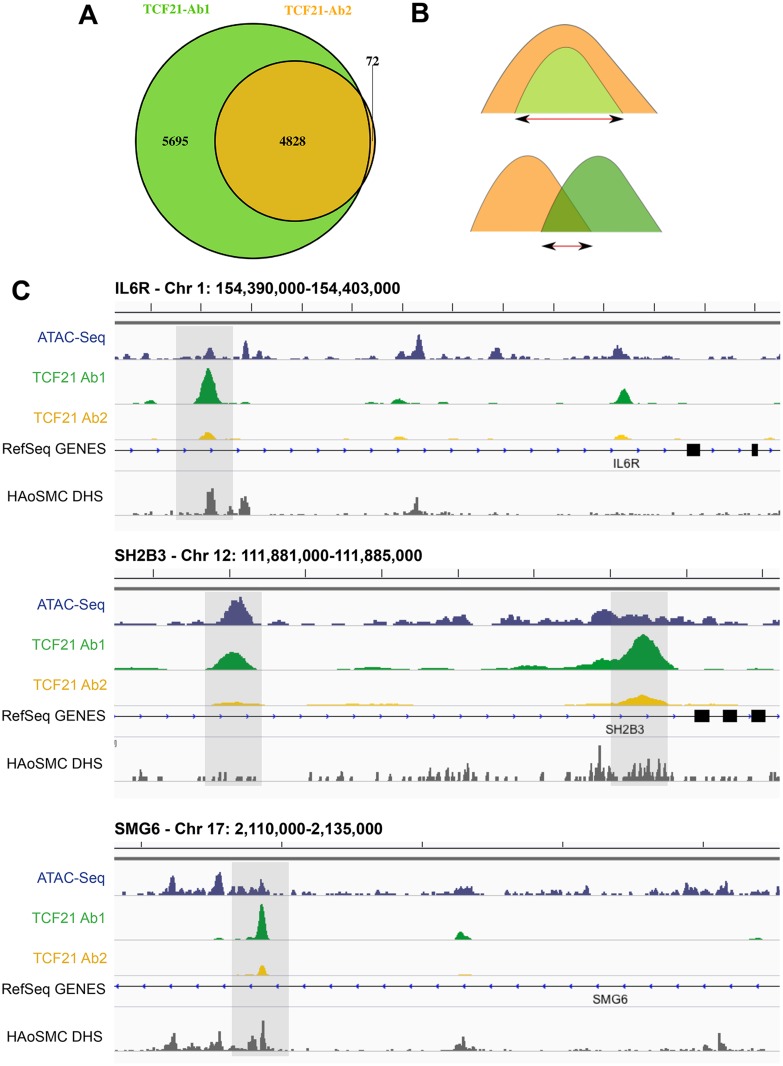
Two TCF21 antibodies show overlapping patterns of TCF21 chromosomal binding. A) Two replicate experiments with Antibody 2 (Ab2) identified 4828 binding sites. All but 72 of these peaks were also identified by similar replicate experiments with Ab1, which recognized an additional 5695 peaks. B) In addition to analyzing data for each of the two antibody ChIP-Seq datasets, we have intersected those identified with both Ab1 and Ab2 (Ab_Shared), with the smaller of the peaks being employed if there was complete overlap of one versus the other, and the region of overlap used if the two peaks shared incomplete overlap. C) High throughput next-generation sequencing reads were aligned to the genome, peaks present in both biological replicates of each of the two antibody precipitations were identified by IDR, and visualized on the UCSC browser [8]. In addition to the TCF21 ChIP-Seq data, also shown are ATAC-Seq data for HCASMC and DNse I hypersensitivity data obtained with human aortic smooth muscle cells (HAoSMC DHS) indicating that TCF21 peaks localize to regions of active chromatin conformation.

Next, we investigated the overlap in genomic regions represented by peaks associated with each antibody precipitation. Results of this analysis also revealed a high degree of overlap between the two peak sets, with all but 72 of the 4900 peaks identified by Ab2 sharing one or more basepairs with peaks identified by Ab1 (Fig 1A). Visualization of peaks with the IGV browser provided further evidence of extensive overlap of peaks, although Ab2 frequently showed decreased peak size compared to Ab1 (Fig 1C). Shown here are TCF21 peak regions in three genes that have been identified as replicated CAD GWAS loci, *IL6R*, *SH2B3*, and *SMG6*. Due to this overlap, as well as the similarities in peak binding patterns described above, we intersected the two datasets to refine the number of peaks to those identified by both antibodies (Ab_shared, Fig 1B) and, unless otherwise noted, employed this data set for the analyses presented below.

### ChIP-Seq peaks are located in regions of open chromatin and identify genes differentially regulated by TCF21

A number of approaches were employed to validate results obtained with ChIP-Seq. First, we investigated the overlap between TCF21 peaks and regions of open chromatin as defined for HCASMC by the assay of transposase accessible chromatin high-throughput sequencing (ATAC-Seq) performed in this laboratory [9] and with ENCODE data for human aortic smooth muscle cells (HAoSMC) as identified by DNase hypersensitivity assay. We found that the TCF21 ChIP-Seq peaks significantly overlap with ATAC-Seq signals (*P*<1.0e-300, fold enrichment = 1.85; S1 Table and Fig 1C). Similarly, the TCF21 ChIP-Seq peaks also significantly overlap with the HAoSMC DHS signals (*P*<1.0e-300, fold enrichment = 4.29; S1 Table and Fig 1C). Second, we performed technical replication with ChIP employing separately isolated chromatin from HCASMC derived from a different donor, with PCR primers flanking a number of TCF21 peaks. In these studies, the chosen genes showed 4- to 90-fold enrichment compared to a select non-target region (S2 Fig).

To investigate whether genes with binding peaks are directly regulated by TCF21, we took advantage of existing co-expression networks to investigate overlap between ChIP-Seq identified target genes and those genes that track with *TCF21* gene expression. We retrieved 2705 co-expression modules, each containing highly co-regulated genes, derived from 108 coexpression networks constructed from many different tissues in human and mouse populations [10–19]. We then performed enrichment analysis between the co-expression modules and genes associated with Ab_shared peaks using Fisher’s exact test. The target genes tended to form coexpression modules, and 296 such modules from 10 coexpression networks were significantly enriched with TCF21 target genes at FDR<0.01 (S2 Table). Vascular endothelial cell and vascular disease related adipose tissue coexpression networks were most strongly associated with TCF21 target genes [20]. Taken together, these data provide evidence that expression of target genes is highly coordinated by *TCF21* and that identified peaks functionally regulate target gene expression.

### Pathway analyses suggest that TCF21 regulates growth factor signaling as well as cell-cell and cell-matrix interactions

To investigate the molecular and cellular processes downstream of TCF21 and possible mechanisms of disease association, studies were conducted to look for over-representation of TCF21 target genes among well annotated regulatory pathways. Here, we analyzed the common peak set (Ab_shared) with the Genomic Regions Enrichment of Annotations Tool (GREAT) [21]. GREAT assigned genes to peaks and queried a number of functional databases with the resulting gene list (Table 2). Evaluation of gene ontology (GO) Molecular Function, GO Biological Process, PANTHER Pathway, and Pathway Commons databases identified terms related to growth factor signaling (“platelet growth factor (PDGF) receptor binding/signaling,” “vascular endothelial cell growth factor (VEGF) signaling”), cell-matrix interactions (“integrin binding,” “cell adhesion”), matrix biology (“extracellular matrix structural constituent”), actin contractile function (“actin filament-based processes,” “actin cytoskeleton”). Mouse phenotype database terms included “abnormal cardiovascular system physiology”, “abnormal blood circulation”, and “abnormal blood vessel morphology”. Importantly, MSigDB Predicted Promoter Motifs ontology identified enrichment among the TCF21 target genes for those with JUN family member binding sites in their promoter regions.

**Table 2:** GREAT pathway terms from analysis of TCF21 Ab_Shared peaks.

Ontology	Term name	Binomial raw P-Value	Binomial FDR Q-Value	Binomial fold enrichment
**GO Molecular Function**	growth factor binding	8.05008e-17	2.73783e-14	3.6097
	extracellular matrix structural constituent	6.08803e-13	1.15030e-10	3.8502
	protein complex binding	8.48872e-12	1.15481e-9	2.3507
	integrin binding	1.03188e-10	1.21014e-8	3.5782
	insulin-like growth factor binding	1.15986e-6	6.26142e-5	4.4830
	glucose binding	8.39566e-5	2.37947e-3	6.9866
	platelet-derived growth factor receptor binding	1.31103e-4	3.53875e-3	4.8780
**GO Biological Process**	response to wounding	3.74287e-34	1.49051e-31	2.2578
	extracellular matrix organization	1.59481e-22	2.85145e-20	4.1471
	cardiovascular system development	3.66444e-22	6.29493e-20	2.2085
	wound healing	2.98700e-21	4.75802e-19	2.2196
	cell adhesion	1.77278e-20	2.72480e-18	2.0695
	vasculature development	2.54347e-19	3.71389e-17	2.4246
	anatomical structure formation involved in morphogenesis	1.57856e-18	2.16090e-16	2.0946
	blood vessel development	2.05280e-18	2.72494e-16	2.4377
	positive regulation of developmental process	6.97076e-18	8.60153e-16	2.0867
	angiogenesis	1.54105e-17	1.77647e-15	2.8009
	actin filament-based process	1.91600e-13	1.42255e-11	2.3146
	actin cytoskeleton organization	3.02899e-13	2.15748e-11	2.3823
**PANTHER Pathway**	Integrin signaling pathway	2.13562e-13	2.98986e-11	2.9133
	Inflammation mediated by chemokine and cytokine signaling pathway	1.29380e-12	9.05663e-11	2.7160
	Interleukin signaling pathway	1.37411e-6	6.41251e-5	2.6025
	Angiogenesis	4.82037e-6	1.68713e-4	2.1136
	Apoptosis signaling pathway	1.28650e-5	3.60219e-4	2.3664
	PDGF signaling pathway	4.44411e-5	1.03696e-3	2.0784
	Ornithine degradation	1.57191e-4	3.14383e-3	10.1958
	VEGF signaling pathway	1.59011e-4	2.78269e-3	2.5901
	Toll receptor signaling pathway	1.13903e-3	1.13903e-2	2.5522
	Hypoxia response via HIF activation	1.62200e-3	1.41925e-2	2.9590
**Pathway Commons**	Integrin family cell surface interactions	8.32211e-48	1.33736e-44	2.2317
	Beta1 integrin cell surface interactions	1.86295e-45	1.49688e-42	2.2082
	VEGF and VEGFR signaling network	9.33799e-39	5.00205e-36	2.1238
	IL3-mediated signaling events	1.72270e-38	6.92093e-36	2.1252
	Alpha9 beta1 integrin signaling events	5.48438e-38	1.76268e-35	2.1100
	Nectin adhesion pathway	9.14983e-38	2.45063e-35	2.1124
	Signaling events mediated by VEGFR1 and VEGFR2	1.10136e-37	2.52840e-35	2.1104
	Endothelins	1.37351e-37	2.75904e-35	2.1027
	PAR1-mediated thrombin signaling events	1.69822e-37	3.03227e-35	2.1057
	Thrombin/protease-activated receptor (PAR) pathway	1.90755e-37	3.06543e-35	2.1044
	Plasma membrane estrogen receptor signaling	1.91002e-37	2.79037e-35	2.1044
	Sphingosine 1-phosphate (S1P) pathway	2.25746e-37	3.02312e-35	2.0999
	EGFR-dependent Endothelin signaling events	3.70783e-37	4.58345e-35	2.1051
	PDGF receptor signaling network	4.30056e-37	4.93643e-35	2.1008
	IFN-gamma pathway	4.33516e-37	4.64440e-35	2.1007
	TRAIL signaling pathway	6.27451e-37	6.30196e-35	2.0837

### TCF21 peaks contain the CAGCTG E-box motif as well as an activator protein-1 (AP-1)-like motif that mediates JUN factor binding

**Fig 2:**
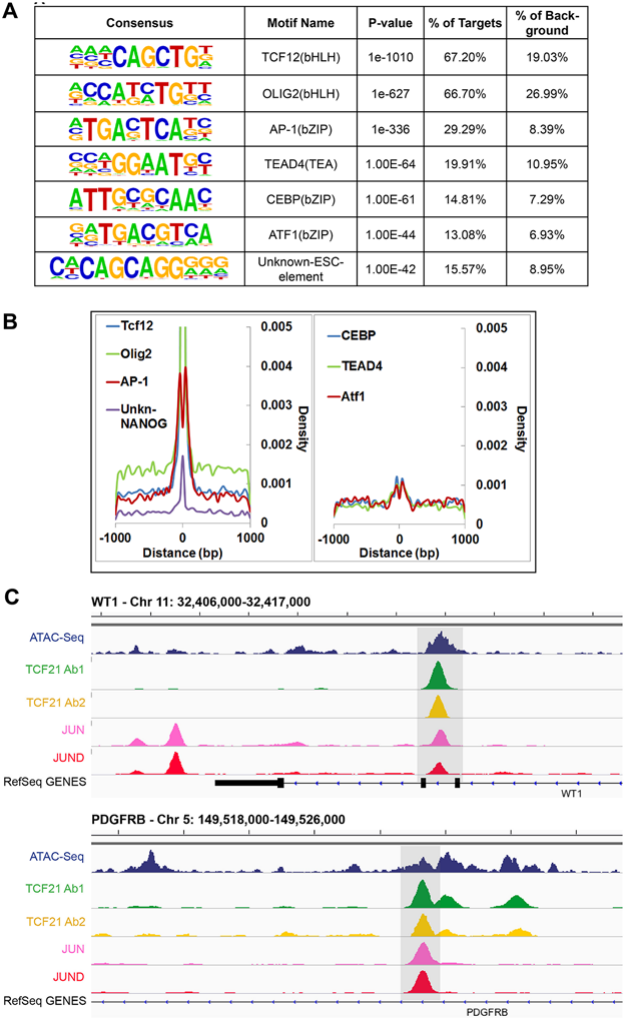
Analysis of peak sequences identifies TCF21 binding motifs as well as motifs for JUN related and other transcription factors that likely cooperate with TCF21. A) HOMER analysis of known TF motif enrichment within TCF21 peaks in the Ab_Shared data revealed several distinct motif families. The bHLH motif CAGCTG is identical to that attributed to TCF12, a known heterodimer partner of TCF21 [26], and a second highly enriched bHLH motif CATCTG is attributed to nervous system TF OLIG2, suggesting that TCF21 can bind either of these two motifs. The bZIP motif TGA(G/C)TCA most closely resembles the binding sequence for TFs within the AP-1/ATF super family. Other motifs found to be enriched in the TCF21 peaks include those mediating binding of TEAD, CEBP, and ATF transcription factor family members, and an unknown element identified by ChIP-Seq with NANOG in human embryonic stem cells (ESC). B) Distribution (density) plots for top 7 motifs from panel A: TCF12, OLIG2, AP-1, unknown-NANOG (left), and CEBP, TEAD4, ATF1 (right). C) TCF21 binds in close proximity to JUN and JUND in a number of loci, including developmentally important *WT1* and *PDGFRB* loci.

The availability of sequence information across a large number of TF binding sites allowed identification of the canonical binding sequence for TCF21. We employed the HOMER and MEME-ChIP algorithms for this task, investigating de novo TF motif enrichment within TCF21 peaks shared between Ab1 and Ab2 [22,23]. This analysis identified the nucleotide sequence CAGCTG in 67.2% of peaks (*P* = 1e-1010) (Fig 2A). This sequence matched the CANNTG sequence that is the common E-box binding motif used by bHLH factors, and is identical to the E-box motif that is known to mediate binding of bHLH partners of TCF21, including TCF12 [24]. An additional E-box motif (CATCTG) was found in 66.7% of peaks (*P* = 1e-627), and identified as identical to the motif recognized by bHLH factor Olig2, likely representing a second motif that is recognized by TCF21. Interestingly, an additional enriched TF binding motif was also identified in approximately 30% of peaks, corresponding to the bZIP motif TGA(G/C)TCA (*P* = 1e-336) that is known to bind the AP-1 family of TFs. Other motifs of interest included those that mediate binding of TEAD and CEBP transcription factor families. Graphing the distribution of these motifs in comparison to the summits of TCF21 peaks suggested that AP-1 and possibly ATF1 factors bind in a bimodal pattern flanking TCF21, suggesting a possible steric binding relationship between TCF21 and these bZip factors (Fig 2B). These motifs likely mediate binding of TFs that cooperate with TCF21 to direct transcriptional programs associated with target genes, as has been characterized for other TFs [25].

### Follow-up ChIP-Seq studies verify over-representation of JUN family member binding at TCF21 target genes

We have previously shown that JUN and other AP-1-related transcription factors transactivate *TCF21*, and disruption of this pathway by disease-associated allelic variation in the related binding site may account in part for the CAD susceptibility observed at this locus [27]. To explore whether JUN factors may also bind in association with TCF21 to co-regulate target genes in arterial smooth muscle cells, we performed ChIP-Seq in HCASMC for JUN and JUND. Samples were processed, sequences aligned to the genome, and peaks called with the same algorithms as for the TCF21 experiments. We quantified the overlap of TCF21 and JUN/JUND binding regions against a background of all regions of open chromatin, with the analyses employing both ATAC-Seq study of HCASMC and DHS study of HAoSMC to define this background. For the analysis with ATAC-Seq regions we found significant overlap of TCF21 with JUN and JUND binding sites (*P*<4.12e-215, fold enrichment = 2.84), and employing the same methods with HAoSMC DHS regions as background, TCF21 overlap with JUN and JUND peaks remained significant (*P*<1.79e-183, fold enrichment = 2.21).

Example genomic regions with overlap of TCF21, JUN and JUND binding are shown for the developmental *WT1* gene and the developmental growth factor *PDGFRB* gene (Fig 2C). This and other labs have shown that WT1 regulates *TCF21* [27,28], but TCF21 binding in the *WT1* locus as demonstrated here is novel and provides support for a bidirectional regulatory interaction between these important developmental factors. Collectively, these analyses reveal common genome-wide binding patterns between TCF21, JUN and JUND, providing strong evidence for the coordinated binding of TCF21 and JUN family members in HCASMC. Taken together with previously published data showing that JUN factors are upstream regulators of *TCF21* transcription, these results suggest a compelling functional link between these TF pathways.

### Functional SNPs mapped to genes targeted by TCF21 are significantly enriched among CAD GWAS SNPs

To look for more functional relationships between TCF21 target gene SNPs and those associated with other CAD genes, further analyses were conducted employing regulatory SNPs (eQTLS) which have been identified through studies in a variety of tissues investigating the genetic basis of gene expression [11,16,29–31]. We retrieved eQTLs from liver, brain, blood, human aortic endothelial cells (HAEC), adipose tissues and collected all the eQTLs/functional SNPs mapped to specific target genes [11–16,29,31–33]. eQTLs/functional SNPs mapped to genes targeted by TCF21 were significantly enriched among SNPs with low CARDIoGRAM GWAS *P*-values (P<0.01) (fold enrichment 1.78 to 2.42, *P* = 1.24e-12 to 3.69e-95 in all eSNP sets tested; Table 3). Additionally, we obtained all the functional SNPs from RegulomeDB (http://www.regulomedb.org/, based on ENCODE) and evaluated them in the context of their functional annotation. Functional SNPs for TCF21 target genes as defined by the RegulomeDB Category I (i.e., SNPs with highest level of evidence that they have functional influence on genes) showed the highest fold enrichment for SNPs with CAD GWAS *P*-values < 0.01 (Category I fold enrichment 2.06; *P* = 1.04e-155). Category II SNPs (SNPs with less functional evidence than Category I) also showed highly significant enrichment for low P-values (fold enrichment 1.42; P = 2.60e-40) (Table 3). All analyses were controlled for LD, with SNPs possessing r^2^>0.3 removed.

**Table 3:** Enrichment of functional SNPs (based on eQTL mapping and ENCODE) with low P-value associations in CARDIoGRAM GWAS for SNPs of TCF21 target genes.

eQTL tissues, ENCODE categories+	No. independent functional SNPs tested in CARDIoGRAM CAD GWAS+	No. functional SNPs reaching P<0.01 in CARDIoGRAM CAD GWAS	No. functional SNPs in TCF21 peak genes	No. functional SNPs of TCF21 peak genes reaching P<0.01 in CARDIoGRAM CAD GWAS	Fold Enrichment of functional SNPs with low CAD association P-value	Enrichment P-value by Fisher's exact test	Enrichment P-value by KS test
eQTL tissues							
Adipose	81223	1488	12362	548	2.42	3.69e-95*	2.19e-27*
Liver	81223	1488	8504	324	2.08	4.17e-38*	5.95e-58*
Blood	81223	1488	11576	381	1.80	1.07e-31*	1.20e-63*
Brain	81223	1488	3924	171	2.38	7.27e-26*	5.99e-24*
HAEC	81223	1488	4889	159	1.78	1.24e-12*	3.30e-83*
ENCODE categories							
1	256788	4907	34338	1354	2.06	1.04e-155*	9.40e-208*
2	256788	4907	46217	1252	1.42	2.60e-40*	<e-300*
3	256788	4907	30429	619	1.06	0.046	<e-300*
4	256788	4907	95580	2413	1.32	8.14e-67*	<e-300*
5	256788	4907	25	0	0	0.38	0.12
6	256788	4907	24	0	0	0.37	5.91e-12*

^+^Filtered using LD cutoff r^2^ >0.3; only SNPs with r^2^ <0.3 are kept.

*Statistically significant passing Bonferroni-corrected P<0.05.

### TCF21 target genes show significant over-representation among GWAS genes associated with CAD

We noted previously that a number of the CAD loci that have reached genome wide significance contain TCF21 peaks (Fig 1C), and reasoned that since TCF21 is a transcription factor one mechanism for its disease association might be through regulation of these other CAD loci. We were thus interested to perform an enrichment analysis with GWAS data to test the hypothesis that TCF21 affects CAD by modulating a larger than expected number of CAD-related genes. Significant enrichment of TCF21 targets among CAD loci would support this hypothesis. To investigate this possibility, we took two complementary approaches to look for over-representation of TCF21 binding regions among CAD GWAS loci. The first approach was based on gene-level overlap by assessing enrichment of TCF21 target genes among candidate genes in the CHD GWAS loci. The second approach was based on SNP-level linkage information by evaluating whether the average linkage disequilibrium (LD) between TCF21 peak SNPs and CAD GWAS loci SNPs is greater than expected by chance. To test the specificity of TCF21 target regions to CAD, we also included additional phenotypes for comparison. The traits/phenotypes that we investigated with both analyses included: *i)* coronary artery disease phenotypes, *ii)* risk factors that are known to be associated with CAD, *iii)* non-atherosclerotic vascular diseases that are not specifically associated with CAD, *iv)* primarily inflammatory disease phenotypes that are known to involve molecular pathways that are also linked to CAD, *v)* disease phenotypes related to tissues where TCF21 is known to not be expressed and which were predicted negative controls. Also, we focused analyses on traits with the largest number of associated variants, with the goal of strengthening the statistical analysis, and primarily employed traits/phenotypes with at least 20 associated variants. Phenotypes or traits that passed *P*<0.05 from both methods were deemed significant, yielding a combined cutoff of *P*<0.0025. Considering ~20 disease sets were tested, this combined statistical cutoff is equivalent to a Bonferroni-corrected *P*<0.05.

In the first analysis, we investigated over-representation of TCF21 binding region genes among CAD locus genes. As a preliminary analysis, to test for possible confounding, we tested whether TCF21 binding is by chance more likely to be near genes mapped for various GWAS phenotypes by running an enrichment analysis between genes linked to TCF21 binding sites and all GWAS genes from the GWAS Catalog [34]. As shown in S3 Table, although there is statistically significant over-representation of Ab2 and Ab_Shared binding site genes among CAD GWAS genes, the fold enrichments are all close to 1, indicating very minor enrichment of overall GWAS signals among the TCF21 targets. We thus assigned genes to TCF21 peaks employing a distance metric of 50 kb (S4 Table), compiled a list of candidate genes for each phenotype/trait, and tested for enrichment of TCF21 target genes among disease/trait candidate genes. Enrichment analyses for chosen phenotypes/traits were conducted using all GWAS genes as background to correct for the slight over-representation of GWAS genes among those that are TCF21 targets. In addition, to correct for any potential bias in the large numbers of GWAS genes for certain traits such as height and CAD, we implemented a permutation strategy by generating 1000 random GWAS gene sets of matching size for each trait to derive permutation-based enrichment *P*-values. The methodology for this permutation-based analysis is provided in the Methods section.

Employing genes in the GWAS catalog associated with CAD phenotypes, enrichment was found for TCF21 target genes among CAD genes compared to a background of all GWAS genes (CAD, 1.34-fold enrichment, permutation *P* = 0.014) and these results did not change substantially with exclusion of lipid trait genes (CAD no lipid, 1.34-fold enrichment, permutation *P* = 0.03) (Table 4). When CARDIoGRAM+C4D data was included in the analysis (CAD extended) the fold enrichment increased to 1.51 (permutation *P*<1.0e-03) and again this did not change substantially with removal of lipid trait variation (CAD extended no lipid, 1.53-fold enrichment, permutation *P*<1.0e-03). We also found that the candidate sets of GWAS genes associated with the CAD related trait platelet number and a disease phenotype related to a dysfunctional immune system, inflammatory bowel disease (IBD), also showed a high degree of enrichment for TCF21 target genes among the GWAS gene sets. At Bonferroni corrected *P*<0.05 (raw *P*<0.0022) in this test, height, CAD extended, CAD extended no lipid, IBD, and platelet phenotypes reached statistical significance. Importantly, we found little evidence of enrichment of TCF21 target genes among GWAS candidate genes for risk factors blood pressure, lipids, and glucometabolic related traits.

**Table 4:** Enrichment of TCF21 target genes from Ab_Shared among GWAS candidate trait genes using all GWAS genes as background and a permutation strategy to correct for the differences in the numbers of GWAS genes between traits.

GWAS traits	TCF21 target genes (Ab_Shared)	No. overlapping genes	No. trait GWAS genes	No. total GWAS genes	Fold enrich-ment	Average fold enrichment in permuted sets	Enrichment P-value (Fisher's)	Permutation-based enrichment P-value (1000 permutations)
Height	2094	118	414	8277	1.41	1	4.72e-07	<1.0e-03
*CAD extended	2094	90	239	8371*	1.51	1.01	4.05e-06	<1.0e-03
*CAD extended no lipid	2094	78	204	8357*	1.53	1	8.97e-05	<1.0e-03
Inflammatory bowel disease	2094	85	232	8277	1.45	1	3.82e-05	<1.0e-03
Platelet	2094	32	75	8277	1.69	1.01	3.08e-04	0.001
Aneurysm	2094	16	35	8277	1.81	1.01	2.46e-03	0.007
Systemic lupus erythematosis	2094	25	64	8277	1.54	1.01	4.97e-03	0.012
CAD	2094	49	145	8277	1.34	1	8.09e-03	0.014
Migraine	2094	11	23	8277	1.89	0.99	5.10e-03	0.02
CAD no lipid	2094	42	124	8277	1.34	1	1.20e-02	0.03
Breast cancer	2094	38	131	8277	1.15	1	1.39e-01	0.117
Schizophrenia	2094	50	193	8277	1.02	1	3.85e-01	0.301
B function glucose	2094	14	57	8277	0.97	1.01	4.80e-01	0.369
Blood pressure	2094	36	138	8277	1.03	0.99	3.72e-01	0.38
Insulin resistance	2094	16	62	8277	1.02	1.01	3.97e-01	0.417
Celiac disease	2094	27	107	8277	1	0.99	4.55e-01	0.446
Triglyceride levels	2094	27	107	8277	1	1.01	4.55e-01	0.455
Ulcerative colitis	2094	34	137	8277	0.98	0.99	5.06e-01	0.5
Kawasaki disease	2094	4	20	8277	0.79	0.99	5.97e-01	0.605
Parkinson's	2094	17	71	8277	0.95	1	5.42e-01	0.645
Macular degen	2094	14	66	8277	0.84	0.99	7.29e-01	0.717
Cholesterol total	2094	22	100	8277	0.87	1.01	7.38e-01	0.756
LDL cholesterol	2094	18	102	8277	0.7	1.01	9.57e-01	0.949

*GWAS catalog genes were supplemented with additional CAD genes identified from CARDIoGRAM+C4D, these genes are not currently included in the GWAS catalog.

**Fig 3:**
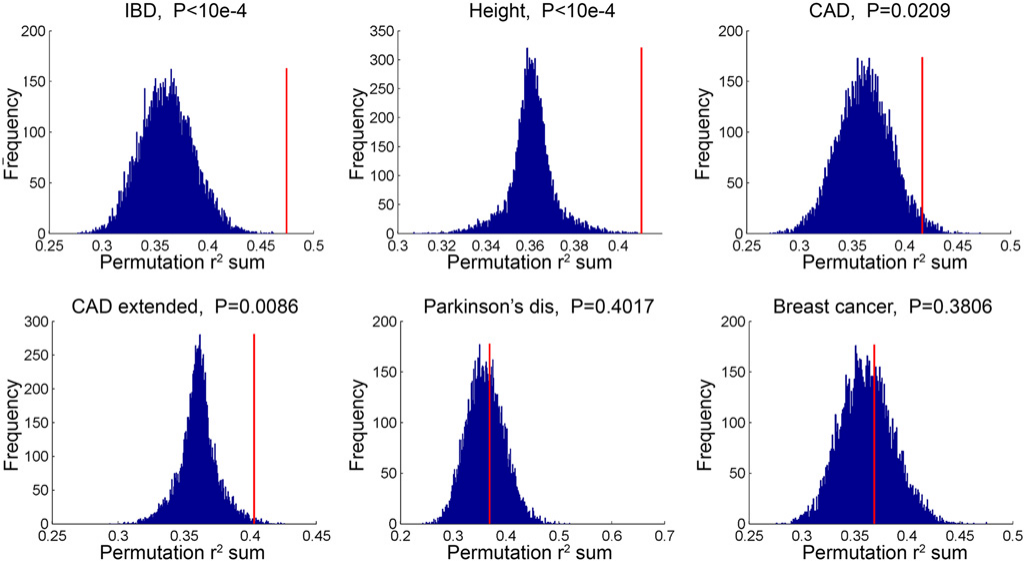
TCF21 target regions contain variation that is in linkage disequilibrium with CAD GWAS variation. SNPs in TCF21 peaks were evaluated for association with GWAS SNPs on the basis of linkage disequilibrium; r^2^ values for each SNP pair were averaged for the GWAS categories inflammatory bowel disease (IBD), height, CAD, CAD plus CARDIoGRAM+C4D (CAD extended), Parkinson’s disease, and breast cancer. To test for enrichment in each category, permutation analysis was utilized to generate distributions of average r^2^ using random sets of SNP pairs and statistical significance was assigned to those categories where fewer than 5% of permutations produced an r^2^ greater than or equal to the true data.

In a second type of analysis conducted at the SNP level, we investigated whether common variants in regions targeted by TCF21 binding tend to demonstrate higher LD with SNPs associated with CAD by GWAS. Such a link would provide additional evidence for the involvement of TCF21 in the genetic pathways that contribute to CAD risk and serve as a complementary approach to the gene-based analysis described above. After pruning SNPs associated with both the CAD loci and TCF21 peaks for LD, we investigated whether SNPs in the TCF21 binding sites were in higher than expected LD with CAD-associated genetic variants compared to random SNPs. A similar analysis was done for other GWAS phenotypes to test the specificity to CAD. For each trait-associated SNP set, permutation analysis was utilized to generate distributions of average r2 using 10,000 random sets of TCF21-GWAS SNP pairs, and statistical significance was assigned to those categories where fewer than 5% of permutations produced an average r^2^ greater than or equal to the true data. Results from this analysis showed that SNPs in TCF21 peaks have significantly greater LD than expected by chance with SNPs for CAD related phenotypes: CAD (permutation *P* = 0.0209) and for CAD Extended (permutation *P* = 0.0086) that analyzed GWAS SNPs plus those from CARDIoGRAM+C4D (Table 5 and Fig 3). With these analyses, the CAD categories without lipid variants were marginally more significant than CAD categories including lipid loci. Interestingly, the greatest enrichment was found for non-CAD phenotypes, including height and IBD as found for the gene enrichment analysis, as well as schizophrenia. With Bonferroni correction for multiple testing these three non-CAD phenotypes reached statistical significance of *P*<0.05. However, when considering the consistent phenotypes between this test and the previous gene level enrichment analysis for GWAS phenotypes, the CAD phenotypes CAD, CAD extended, CAD nolipid, CAD extended nolipid, as well as height, IBD, and platelet number were found to be significant at *P*<0.05 in both tests, yielding a combined *P*<0.0025 which is equivalent to Bonferroni-corrected *P*<0.05.

**Table 5:** Analysis of linkage disequilibrium between SNPs in GWAS loci of select phenotypes and TCF21 peak regions for chosen phenotypes.

Traits	Average LD Values	GWAS SNPs	GWAS SNPs having R2 with TCF21 SNPs	TCF21 SNPs before LD pruning	TCF21 SNPs with LD pruning	P-value with 10000 permutations
**Inflammatory bowel disease**	0.474119347	117	79	535	169	P<10e-4
**Height**	0.410534747	321	218	1143	432	P<10e-4
Schizophrenia	0.45038231	120	83	450	167	0.0004
Ulcerative colitis	0.446449065	82	55	401	115	0.0041
* **CAD extended nolipid**	0.406510724	223	150	718	296	0.0052
* **CAD extended**	0.402737992	229	152	735	305	0.0086
Insulin resistance	0.461195794	51	31	132	58	0.0104
**CAD nolipid**	0.41715869	122	76	316	150	0.0197
**CAD**	0.416045058	126	78	328	155	0.0209
Blood pressure	0.419870421	111	65	322	123	0.0263
**Platelets**	0.426035533	61	49	251	95	0.0284
Triglycerides	0.417955674	52	37	202	78	0.0695
Aneurysm	0.436847165	25	17	103	35	0.0896
Macular degen	0.423410192	45	25	80	45	0.0909
Kawasaki disease	0.449048952	18	11	66	18	0.112
LDL cholesterol	0.401895683	78	47	218	99	0.1148
Cholesterol total	0.388545716	78	55	279	116	0.1798
B function glucose	0.390955994	48	33	150	61	0.2263
Celiac disease	0.374576605	56	30	172	54	0.3688
Breast cancer	0.368659838	131	81	373	141	0.3806
Parkinson disease	0.368841594	67	42	294	81	0.4017
Migraine	0.361138473	133	86	357	165	0.4966
Systemic lupus erythematosus	0.335187216	72	49	260	92	0.7776

*GWAS catalog genes were supplemented with additional CAD genes identified from CARDIoGRAM+C4D, which are not currently included in the GWAS catalog. Traits with bold text are those significant at P<0.05 in both the GWAS gene enrichment analysis shown in Table 4 and the current analysis.

### TCF21 target CAD loci are enriched for genes involved in vascular wall biological processes

To investigate possible functional relationships among the TCF21 target CAD loci, we evaluated the degree of representation of these genes in annotated functional pathways. For this assessment, we used the genes assigned to TCF21 peaks with the DAVID algorithm. We first investigated the pathways that were identified individually for the TCF21 target CAD loci and the full list of CAD loci, quantifying enrichment of genes in loci with those gene ontology (GO) terms annotated for the “biological processes” category. For the TCF21 target CAD genes (57% of the total CAD genes), the top terms that reached significance were disease ontology terms related to migration, including “regulation of cell migration,” “regulation of locomotion,” as well as those related to cell division, “regulation of proliferation,” and metabolism, “regulation of phosphorous metabolic and signaling processes,” regulation of protein amino acid phosphorylation” (S5 Table). Only 3 of the top 20 terms were related to lipids or lipid metabolism. By contrast, when all of the CAD genes were employed in the analysis, there was a preponderance of top terms related to lipid metabolism, “lipid homeostasis,” “cholesterol transport,” and “cholesterol efflux,” with 17 of the top 20 GO terms related to lipids (S6 Table). Also, we performed the analysis with TCF21 target CAD genes being analyzed with the background of all CAD genes (S7 Table). Although this resulted in a decreased number of significant results, this analysis identified cellular traits, including “actin filament based processes,” and “actin cytoskeleton organization,” which represent a smooth muscle signature, and “transcription factor binding,” “intracellular signaling cascade,” and “regulation of DNA binding,” which are all highly suggestive of fundamental cell signaling functions of smooth muscle cells in the vessel wall, possibly related to disease-related processes.

**Fig 4:**
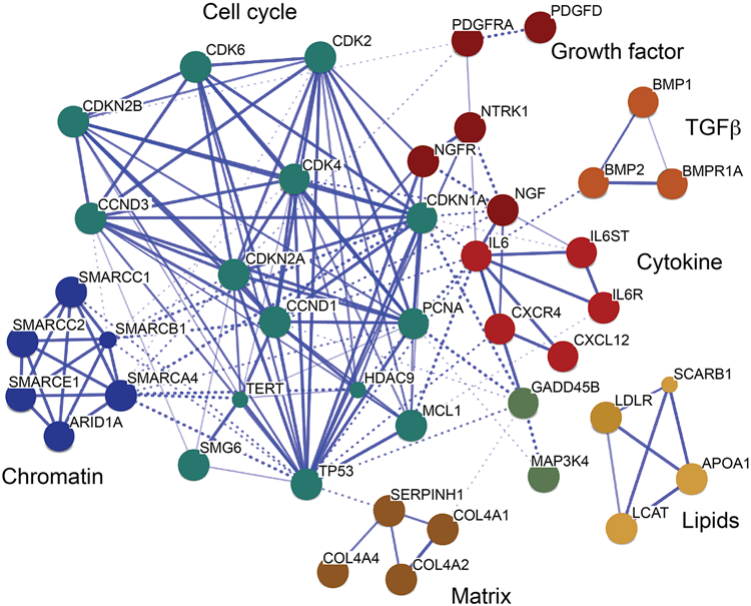
A CAD gene network identified among TCF21 target genes. Genes in CAD-associated loci that have TCF21 peaks identified by ChIP-Seq were assembled into a network, with incorporation of additional non-associated genes to promote the connectivity shown here. This transcriptional network was created by STRING [35]. Many additional CAD genes downstream of TCF21 were not incorporated into the network, due to an overall lack of functional annotation for these loci.

Further, we investigated the interaction of TCF21 peak CAD genes with other genes with the STRING algorithm that integrates protein-protein interaction, text mining and genomic data to link candidate genes into a related network [35]. For this analysis, we employed STRING to generate a network seeded with CHD genes within 50 kb of a TCF21 peak, and then further modified that gene set by including related interacting genes and removing non-connected genes. The resulting network showed clusters of genes associated with cell cycle regulation, extracellular matrix, lipid metabolism, cytokine signaling and growth factor signaling, suggesting that these processes that are relevant to SMC biology are involved in the pathogenesis of CAD (Fig 4).

## Discussion

We focused ChIP-Seq experiments on smooth muscle cells based on previous findings demonstrating the role of TCF21 in coronary vascular development during embryogenesis and its relevance for the CAD phenotype. During development, TCF21 is expressed in SMC progenitors but not in endothelial or other cell types present in vascular disease lesions [2,6]. Vascular SMC response to growth factor signaling is increasingly recognized as an important component of the vascular response to injury where atherosclerotic disease progression is characterized by SMC undergoing phenotypic modulation, changing from a quiescent cell expressing contractile lineage markers to a proliferative, migratory, matrix synthesizing cell that is characteristic of the disease state [7]. In order to better understand the role of TCF21 in CAD progression, we utilized an in vitro model of undifferentiated SMC phenotype based on serum stimulation for the studies presented here [36]. Analysis of TCF21 peaks revealed enrichment for a number of pathways related to the known vascular developmental role of TCF21, as well as growth factor stimulation and signaling, cell-matrix interactions, matrix composition, and actomyosin contractile function. The query of relevant mouse databases revealed enrichment within terms related to vascular phenotypes, including “abnormal blood vessel morphology” and “abnormal blood circulation.” These data suggest that TCF21 directly regulates genes centrally involved in pathways that mediate pathologic SMC processes. Given the association of TCF21 with CAD, our findings thus provide novel evidence supporting the role of SMC in the etiology of disease processes within the vascular wall.

We provide evidence for cooperation of TCF21 with other transcription factors through the finding of other TF binding motifs in the ChIP-Seq peak sequences. We found evidence for enrichment of JUN (AP-1) and several other bZip factor binding motifs, including CEBP and ATF1, in the TCF21 peaks. There was also enrichment for binding sites recognizing the TEAD family of transcription factors that have been linked to signaling through the Hippo pathway that regulates developmental and cancer related cellular processes [37]. We found the identification of JUN related binding sites particularly interesting, but given that these AP-1 motifs are among those that have been found to be commonly enriched in datasets for which they were not the targeted transcription factor [38], we were compelled to perform ChIP-Seq studies for JUN and JUND in the HCASMC. These studies confirmed the in silico prediction, revealing significant enrichment of binding sites found for both of these TFs in genes that also contain TCF21 peaks. For a number of genes, TCF21 and JUN factors were found to bind in close proximity with evidence for JUN factor binding flanking the TCF21 binding site. As noted above, the pathway analysis of TCF21 target genes has identified enrichment for signaling through growth factor binding pathways, including PDGF. PDGF signaling related to smooth muscle cell proliferation is known to involve JUN TFs [39], and our previous studies have shown that PDGF and JUN are upstream regulators of *TCF21* expression [27]. These findings, when coupled with the observation that TCF21 promotes cellular proliferation and dedifferentiation in skeletal muscle cells [40], provide new insight into the relationship between TCF21 and certain growth factor pathways involving AP-1, and suggest that TCF21 is both regulated by and cooperates with this TF family.

These ChIP-Seq studies have identified a number of other CAD associated loci and genes that are bound by TCF21, identifying a disease related transcriptional network. To investigate the hypothesis that TCF21 targets a greater than expected number of CAD-related genes we performed enrichment analysis at both the gene and SNP level with GWAS data. These studies identified a greater than expected number of TCF21 targets among CAD GWAS genes, and showed that TCF21 peak region SNPs are in greater linkage disequilibrium with CAD GWAS SNPS than expected. Since the ChIPseq data was generated in HCASMC, a cell type found in the coronary artery, the results of CAD loci enrichment is consistent with the hypothesis that TCF21 regulates a greater than expected number of CAD genes in the vessel wall and that its effect on disease risk may be due in part to its regulation of the network of TCF21 target CAD associated genes. Consistent with TCF21 CAD target genes being active primarily in the vascular wall, we saw no difference in enrichment between the CAD analyses with and without lipid genes or SNPs being included in the analysis, and no enrichment was found for vascular risk factor traits “LDL cholesterol” and “Total cholesterol.” The enrichment for “Platelets” is not surprising given the known association of platelet number with vascular disease events, and this association could explain some portion of the *TCF21* risk for CAD [41–43]. Human genetic epistasis studies are required to validate these putative gene-gene interactions between TCF21 and the other CAD genes in the network, but further study of the functional links among validated TCF21 target CAD genes should provide an opportunity for further dissecting the molecular basis of disease risk.

Surprisingly, both the gene- and SNP-level enrichment GWAS analyses also found TCF21 target genes to be over-represented among those associated with height and immune related IBD. Although SMC would seem unlikely to be involved in the height and IBD phenotypes, we speculate that enrichment of TCF21 target genes within these other phenotypes is real and reflects overlapping biology between the physiology/pathophysiology of these phenotypes and that of CAD. For instance, short stature has been repeatedly linked to an elevated risk for CAD, suggesting the presence of a substantial overlap in the biological pathways that determine height as well as the risk of CAD [44–46]. In support of a causal link between shorter stature and higher risk of CAD, a recent Mendelian Randomization study found a graded relationship between a genetic risk score of 180 height raising alleles identified by the GIANT consortium and the risk of CAD [47,48]. Furthermore, pathway analysis of genes near or within the height loci identified enrichment of several canonical pathways involved in both growth-development and atherosclerosis [48]. It is also not surprising to observe enrichment for immune related pathways that mediate IBD given inflammation has been extensively linked to atherosclerosis at an experimental level [49–51]. These findings are also consistent with our pathway analyses of TCF21 related CAD genes (S7 Table). We note that the genes that contribute to the enrichment in these phenotypes are unlikely to be regulated by TCF21 given TCF21 is not associated with these phenotypes in GWAS. Instead, we suspect this enrichment reflects functional roles of related networks in traits that involve multiple other tissues.

Our analyses provide multiple lines of circumstantial evidence supporting a role for the identified TCF21 related transcriptional network in the etiology of CAD. First, the TCF21 target genes are highly enriched for molecular processes with established relationships to CAD, such as growth factor binding, cell adhesion, vascular development, inflammation, and PDGF signaling. Second, the TCF21 target genes are enriched for established CAD genes identified through GWAS. Third, the functional SNPs of TCF21 target genes are enriched for, and tend to be in significant LD with, SNPs associated with CAD in GWAS. Fourth, the finding that the TCF21 transcriptional network is enriched in other traits is consistent with a biological program that contributes to a number of disease-relevant tissue-specific cellular processes. However, further work is required to firmly establish our hypothesis that the TCF21 transcriptional gene network contributes to CAD risk. If TCF21 binding to other CAD loci is important for the disease risk mechanism, one may be able to identify epistatic effects between polymorphisms in or near TCF21 and other CAD SNPs in the TCF21 target regions. At an experimental level it will be important to link TCF21 binding sites in target loci to function of the causal variant and the causal gene, and to investigate how TCF21 binding may regulate the causal mechanisms at other CAD loci. Finally, it will be important to functionally assess the predicted TCF21 gene network by perturbation in vitro and in vivo with experimental cell culture and animal models. Taken together, these approaches may provide significant new insights into how a disease associated transcription factor may work through other disease-associated loci to modulate disease risk.

## Methods

### HCASMC culture and TCF21 knockdown

Human primary Coronary Artery Smooth Muscle Cells (HCASMCs, #CC-2583, Lot No. 200212) were purchased from Lonza (Allendale, NJ, USA) and cultured in SmGM-2 Smooth Muscle Growth Medium-2 including hEGF, insulin, hFGF-B and FBS, but without antibiotics (Lonza, #CC-3182) for 3 passages. Technical replicate samples were cultured from the same stock vial but harvested 24h apart. The crosslinked nuclei of all replicates were thereafter processed in parallel.

### TCF21 chromatin immunoprecipitation and next generation sequencing (ChIP-Seq)

Ten million HCASMC cells per condition were crosslinked for 10 min with 1% formaldehyde. Cells were lysed for 10 min on ice (10 mM Tris pH8.0, 10 mM NaCl, 0.2% NP-40). Nuclei were then lysed for 10 min on ice (50 mM Tris pH 8.1, 10 mM EDTA, 1% SDS). Crosslinked chromatin was sheared for 3x5 min by sonication using a Bioruptor (Diagenode, Denville, NJ, USA) and pre-cleared with 10 μ g anti-rabbit IgG pre-immune serum (DAKO, Carpintera, CA, USA, #X0903, Lot. No. 25509) using protein G sepharose beads (Roche Diagnostics, Indianapolis, IN, USA, #11 243 233 001). TCF21 ChIP was performed overnight in IP dilution buffer (20 mM Tris pH 8.1, 2 mM EDTA, 150 mM NaCl, 1% Triton X100, 0.01% SDS), using 14 μ g anti-TCF21 (Sigma-Aldrich, St. Louis, MA, USA, #HPA013189, Lot. No. R02939 (Ab1) or Abcam, Cambridge, UK, #ab49475, Lot No. 153899 (Ab2)) or anti-rabbit IgG respectively. JUN and JUND ChIP was performed with Santa Cruz Biotechnology sc-1694 and sc-74 respectively. Beads were washed twice with buffer 1 (20 mM Tris pH 8.1, 2 mM EDTA, 50 mM NaCl, 1% Triton X100, 0.1% SDS), once with buffer 2 (10 mM Tris pH 8.1, 1 mM EDTA, 250 mM LiCl, 1% NP40, 1% sodium deoxycholate monohydrate) and twice with TE buffer. Bound chromatin was eluted from the beads twice with 100 mM NaHCO3 containing 1% SDS. After reverse-crosslinking, RNaseA and proteinase K digestion, chromatin was cleaned up using the Qiagen PCR purification kit (Qiagen, Crawley, UK).

Follow-up confirmatory ChIP was performed with immunoprecipitated and reverse-crosslinked chromatin from HCASMC that was prepared as described above. Quantitative real-time PCR (ViiA 7, Life Technologies) was performed with primers specific for ChIP-Seq peak sequences using SYBR Green (Applied Biosystems) assays and fold enrichment was calculated by measuring the delta Ct—delta Ct IgG. Melting curve analysis was also performed for each ChIP primer pair.

Original data has been deposited at GEO, accession number: GSE61369.

### ChIP-Seq peak identification and target gene mapping

Sequence data were aligned to the reference genome (hg19) using BWA [52]. In the case of TCF21, we identified peaks present in both biological replicates of each of the two antibody precipitations by using Irreproducible Discovery Rate analysis, or IDR, a method extensively used by the ENCODE project [8]. Here, we followed the workflow developed by ENCODE, using MACSv2 to identify peaks against an IgG ChIP-Seq control [8]. Parameters for peak calling and IDR thresholds were set to values recommended by the ENCODE pipeline. We analyzed each antibody composite dataset individually, and also intersected these two datasets to create a dataset with minimal number of peaks and minimal peak length (Fig 1). In the case of JUN and JUND, we used MACSv2 to call peaks against an IgG control using default parameters and selected the top ten thousand peaks (ranked by *Q*-value) for subsequent analysis. We utilized the Genomic Regions Enrichment of Annotations Tool (GREAT) [21] to analyze the detected peaks of each TF, with the parameter “Association rule settings” set to single nearest gene within 50 kb. We also independently identified genes within 50 kb of TF binding sites using the BEDTools software suite [53]. Genes obtained from each method were designated TF target genes.

### Assessment of co-expression among the predicted TCF21 targets

To more broadly assess the co-expression patterns among the TCF21 target genes, we retrieved 2,705 co-expression modules derived from 108 coexpression networks constructed from many different tissues in human and mouse populations [10–19]. The enrichment analysis between the co-expression modules and the target genes was evaluated using Fisher’s exact test.

### ATAC-sequencing

ATAC-Seq was performed with slight modifications to published protocol [9]. Briefly, human coronary artery smooth muscle cells (passage 5–6) were cultured in normal media until about 75% confluence. Approximately 5x10e4 fresh cells were collected by centrifugation at 500 x g and washed twice with cold 1X phosphate buffered saline. Nuclei were extracted with cold lysis buffer containing 10mM Tris-HCl, pH7.4, 10mM NaCL, 3mM MgCl2, 0.1% IGEPAL and nuclei were resuspended in transposition reaction buffer containing Tn5 transposases (Illumina Nextera). Transposition reactions were incubated at 37 C for 30 minutes, followed by DNA purification using Zymo DNA Clean-up and Concentration Kit. Libraries were initially PCR amplified using Nextera barcodes and High Fidelity polymerase (NEB). The number of cycles was empirically determined from an aliquot of the PCR mix, by calculating the Ct value at 25% maximum Rn for each library preparation. The final amplified library was again purified using the Zymo DNA Clean-up and Concentration kit, and the DNA was evaluated by TBE gel electrophoresis and quantitated using Bioanalyzer, nanodrop, and quantitative PCR (KAPA Biosystems). Libraries were multiplexed and paired-end 50bp next generation sequencing was performed using an Illumina HiSeq 2500. Raw FastQ files were evaluated using a modification of the FastQC pipeline to generate per base and per sequence level summary statistics. Libraries that achieved consistent high quality scores from this tool were aligned to the human genome (hg19) using Bowtie2 and transposase sensitive regions were called using MACS, and bigwig files were generated [9].

### De novo and known motif enrichment

To discover DNA binding motifs enriched among TCF21 peaks, HOMER findMotifsGenome.pl script was used to search for the known TRANSFAC motifs and to create de novo motifs from 4852 TCF21 ChIP-Seq binding regions (Ab_Shared regions) [22]. All software parameters were set to default values, with the addition of the “-size given” command to define the width of each peak from the data rather than a constant value. Motifs discovered by HOMER were validated with MEME-ChIP [23] with a maximum motif length of 10. The motifs identified by MEME-ChIP were further compared to the binding motifs of known transcription factors. Density plots of motifs were created as follows. TCF21 summits were defined using MACS with default parameters for the collection of 4852 TCF21 ChIP-Seq binding regions. Motif distribution plots were made using HOMER annotatePeaks.pl script and centering on the locations of TCF21 summits. TRANSFAC matrices for the top HOMER known-motif outputs (TCF12, OLIG2, AP-1, unknown-NANOG, CEBP, TEAD4, ATF1) were used for the scan of TCF21 summit locations in the human GRCh37/hg19 genome. Scanning for motifs was performed using annotatePeaks.pl with the following parameters: hg19-size 2000-hist 20.

### TCF21, ATAC-Seq and HAoSMC DHS peak overlap analysis

We retrieved the HAoSMC DHS data from the ENCODE project and selected peaks with *P*<0.05 as true HAoSMC DHS signal (n = 121731). The overlaps among TCF21 binding regions, ATAC-Seq, and HAoSMC DHS peaks were evaluated through one-sided Fisher exact test. In the overlap between TCF21 ChIP-Seq and ATAC-Seq peaks, we only focused on the ATAC-Seq signals with high-confidence fold-enrichment (mfold>200).

### TCF21 and JUN/JUND peak overlap analysis

We tested co-activity between TCF21 and the AP-1 factors JUN and JUND by evaluating the level of overlap (using BEDTools) between ChIP-Seq binding regions corresponding to each TF. Peaks from each dataset were first reduced to those that overlap with open chromatin as identified with our HCASMC ATAC-Seq or ENCODE HAoSMC DHS data. In each analysis, a one-sided Fisher’s exact test was used to determine whether the observed peak overlap between TCF21 and JUN or between TCF21 and JUND was statistically greater when compared against a background of all HCASMC ATAC-Seq or HAoSMC DHS peaks.

### Analysis of enrichment of association of functional SNPs in TCF21 target loci among functional SNPs of CAD loci

We utilized the publicly available summary level association results (~2.5 million HapMap SNPs) of the CARDIoGRAM consortium involving 22,233 CAD cases and 64,762 controls to further analyze the relevance of the TCF21 target genes to CAD [54]. We explored whether the subset of functional SNPs located in TCF21 target genes had a higher proportion of low *P*-values of association (*P*<0.01) with CAD when compared to the proportion of low *P*-values observed for all functional SNPs examined in CARDIoGRAM, using a Fisher's exact test. The distribution of the *P*-values of these two sets of SNPs was also tested using a one-sided Kolmogorov-Smirnov (KS) test. For this analysis, we identified 189,002 functional SNPs (eSNPs) through eQTL experiments involving liver, brain, blood, human aortic endothelial cells (HAEC), and adipose tissues [11–16,29,31–33,55,56]. In the second approach, we identified 1,201,481 potentially functional SNPs through the ENCODE-based RegulomeDB (http://www.regulomedb.org/). Functional SNPs from RegulomeDB were further classified into various categories based on multiple measures of functional implication according to the database, with category 1 SNPs having the highest number of independent functional measures. TCF21 target genes were first mapped to their associated eSNPs or RegulomeDB SNPs and then compared with the corresponding background functional SNPs of all genes for enrichment of low *P*-value associations with CAD. For both analyses, LD structure was addressed by pruning the eQTL and ENCODE SNPs. This was done by removing those with r^2^ > 0.3 to derive independent SNPs.

### Analysis of enrichment for CAD related and other sets of GWAS genes among TCF21 targets

To investigate possible over-representation of TCF21 binding regions among CHD GWAS loci, we conducted analyses at both the gene and variant level, looking for enrichment of TCF21 target genes among CHD GWAS loci, and for enrichment of average linkage disequilibrium between TCF21 peak SNPs and GWAS loci SNPs.

We first assessed whether there was a spurious association of TCF21 target genes with CAD due to confounding, by looking for an over-representation of these genes among those associated with GWAS loci genes in general. For this analysis, we downloaded the NHGRI GWAS catalog and extracted candidate GWAS genes for all diseases or phenotypes curated in the catalog. Enrichment of candidate GWAS genes among the TCF21 target genes located in the TCF21 peaks was estimated using one-sided Fisher’s exact test. We then looked for an over-representation of CAD candidate genes discovered thorough recent GWAS studies among genes targeted by TCF21. The list of CAD candidate genes we considered (“CAD”) was also collated from the NHGRI GWAS catalog and includes all genes assigned to a SNP in the catalog for phenotypes relevant to CAD including "coronary heart disease", "myocardial infarction", and "coronary artery calcification". For a second category (“CAD extended”) this list was supplemented with genes identified through the CARDIoGRAMplusC4D study, which derive from analysis of Metabochip data and for this reason have not been included in the GWAS catalog [1,34]. Lipid metabolism genes were removed from each of these categories to create lists “CAD no lipid” and “CAD extended no lipid.” We compared the proportion of CAD genes observed among all GWAS catalog phenotypes to the proportion of CAD genes observed among the subset of TCF21 target genes using one-tailed Fisher’s exact test.

For both GWAS enrichment methods we also included additional phenotypes as described in the Result section to test the specificity of CAD signal enrichment in TCF21 targets. These phenotypes included cholesterol measures related to CAD (LDL cholesterol, total cholesterol, triglycerides), diseases known to be associated with dysfunction of the arterial vessel wall or myocardial infarction (aneurysmal conditions involving either the root, the thoracic, or the abdominal aorta, or intracranial vessels (aneurysm), migraine, high blood pressure, platelet number, and Kawasaki's disease), height, which has been associated with CAD, glucometabolic related phenotypes insulin resistance (insulin resistance measures), β-cell function (B function glucose), diseases associated with a dysfunctional immune system (IBD), systemic lupus erythematosus, celiac disease), neuropsychiatric disorders (schizophrenia and Parkinson’s disease), breast cancer and macular degeneration which are well represented in the GWAS catalog but unlikely to be associated with CAD.

To correct for any potential bias in the large numbers of GWAS genes for certain traits such as height and CAD, we implemented a permutation strategy by generating 1000 random GWAS gene sets of matching size for each trait to derive permutation-based enrichment P-values. For example, there are 414 GWAS genes for height, and we generated 1000 gene sets each containing 414 randomly selected GWAS genes from the pool of 8277 GWAS genes in the GWAS catalog. In addition to the enrichment P-value obtained with Fisher’s exact test, (Ptrait) for the observed GWAS gene set of each trait we also calculated 1000 enrichment P-values from the 1000 random gene sets (Prandom1, Prandom2,…, Prandom1000). Ptrait was then compared against the 1000 P-values from the random gene sets to derive a permutation-based enrichment P-value as defined by (the number of Prandom from the random sets that are smaller than Ptrait)/1000. This was done for each of the CAD phenotypes and each additional phenotypes/traits to correct for the different numbers of GWAS genes between phenotypes.

For the TCF21-GWAS LD analysis, we developed a custom pipeline to assess the linkage between variants in TCF21 peaks and variants associated with disease by GWAS. The same GWAS traits/phenotypes were investigated in this analysis as for the gene enrichment analysis. SNPs were identified from the GWAS catalog, where we excluded any GWAS studies that did not contain a cohort of European descent. TCF21 peak SNPs were defined as those 1000 Genomes phase 1 variants that are polymorphic in Europeans and overlap with a TCF21 peak [57]. We used data from the HapMap project (April 2009 release #27) restricted to the CEU population, as well as in-house LD calculations among European SNPs in 1000 Genomes, to assign a linkage disequilibrium r^2^ value to TCF21—GWAS SNP pairs [58]. In the instance where HapMap and 1000 Genomes r^2^ values for a given SNP pair did not agree, we used the average of the two. Using custom software developed in the R language, we calculated the average of all *r* ^*2*^ values between TCF21 peak—trait SNPs pairs, which was denoted as the “true average” for the association between TCF21 peak SNPs and that particular trait. We then computed 10,000 null averages, each time permuting the trait labels in the GWAS catalog and determining the r^2^ average between TCF21 peak SNPs and an equal number of SNPs now incorrectly associated with the trait of interest. In this analysis, if a TCF21 peak SNP was in LD with multiple GWAS SNPs, only the maximum r^2^ value of these associations was taken into calculating the r^2^ average for a given trait. This filter ensured that each TCF21 SNP mapped onto at most one GWAS trait SNP, although a GWAS trait SNP was allowed to map onto multiple TCF21 SNPs.

### Pathway and network analysis of TCF21 target genes

For pathway analysis, the DAVID algorithm [59] was employed with Gene Ontology (GO) terms to annotate the functions of the TCF21 target genes in relation to “biological processes.” Enrichment analysis was carried out in DAVID using default settings. TCF21 target CAD genes and the full list of CAD genes were assessed individually and compared, and the TCF21 target CAD genes were also evaluated in the context of a background composed of all CAD loci genes. For network analysis of TCF21 target CAD genes, those loci associated with CAD at FDR< 0.05 were identified, and genes linked to these loci in the original publications by distance from the lead SNP, eQTL, or allele-specific expression were collated. This gene list was employed with the STRING algorithm [35] to search protein-protein interaction and other types of databases to develop a molecular interaction network.

## Supporting Information

S1 Fig Similar spatial distribution of TCF21 peaks identified by each of two TCF21 antibodies. Distribution of TCF21 peaks identified by Ab1 (A) and Ab2 (B) in relation to transcription start site (TSS). Distribution of TCF21 peaks identified by Ab1 (C) and Ab2 (D) in relation to structural gene features.
(TIF)

S2 Fig Individual ChIP assays verify ChIP-Seq findings. ChIP was performed with separately isolated chromatin from HCASMC derived from a different donor, employing PCR primers flanking a number of TCF21 peaks.
(TIF)

S1 Table Representation of TCF21 ChIP-Seq peaks in regions of open chromatin. (PDF)

S2 Table Enrichment of coexpression modules in TCF21 target genes. (PDF)

S3 Table Enrichment of candidate disease GWAS genes among the target genes of TCF21. (PDF)

S4 Table Coordinates of TCF21 binding sites in CHD GWAS genes. (PDF)

S5 Table GO term enrichment for CAD genes associated with TCF21 peaks. (PDF)

S6 Table GO term enrichment for all CAD genes. (PDF)

S7 Table GO term enrichment for TCF21 CAD genes analyzed with background of all CAD genes. (PDF)
