# Supplementary figures and images for: Characterization of TCF21 Downstream Target Regions Identifies a Transcriptional Network Linking Multiple Independent Coronary Artery Disease Loci
Source: PLoS Genet. 2015 May 28;11(5):e1005202. doi: 10.1371/journal.pgen.1005202 (PMC4447360; doi:10.1371/journal.pgen.1005202)

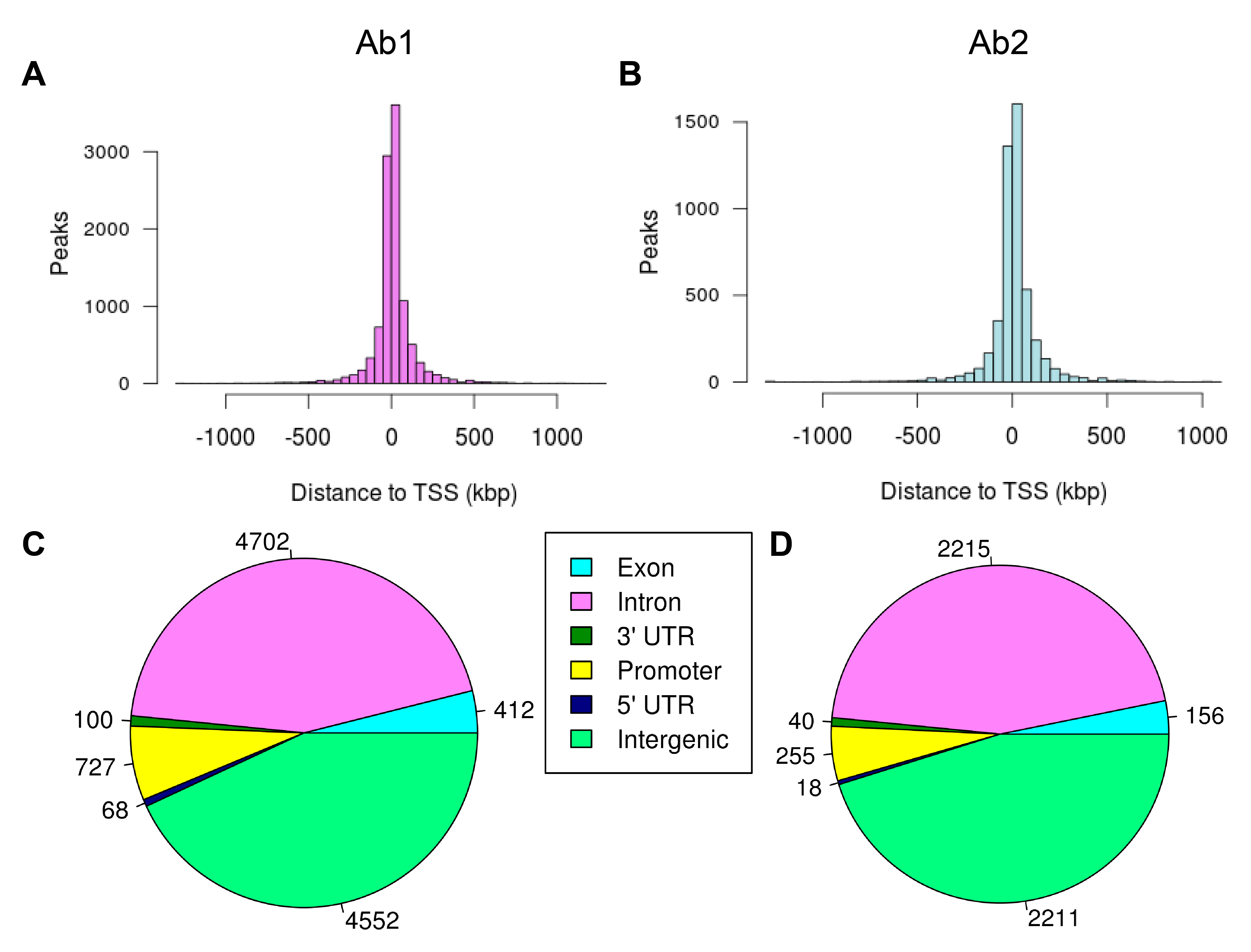

Supplement: S1 Fig — Distribution of TCF21 peaks identified by Ab1 (A) and Ab2 (B) in relation to transcription start site (TSS). Distribution of TCF21 peaks identified by Ab1 (C) and Ab2 (D) in relation to structural gene features. (TIF) [file pgen.1005202.s001.tif]

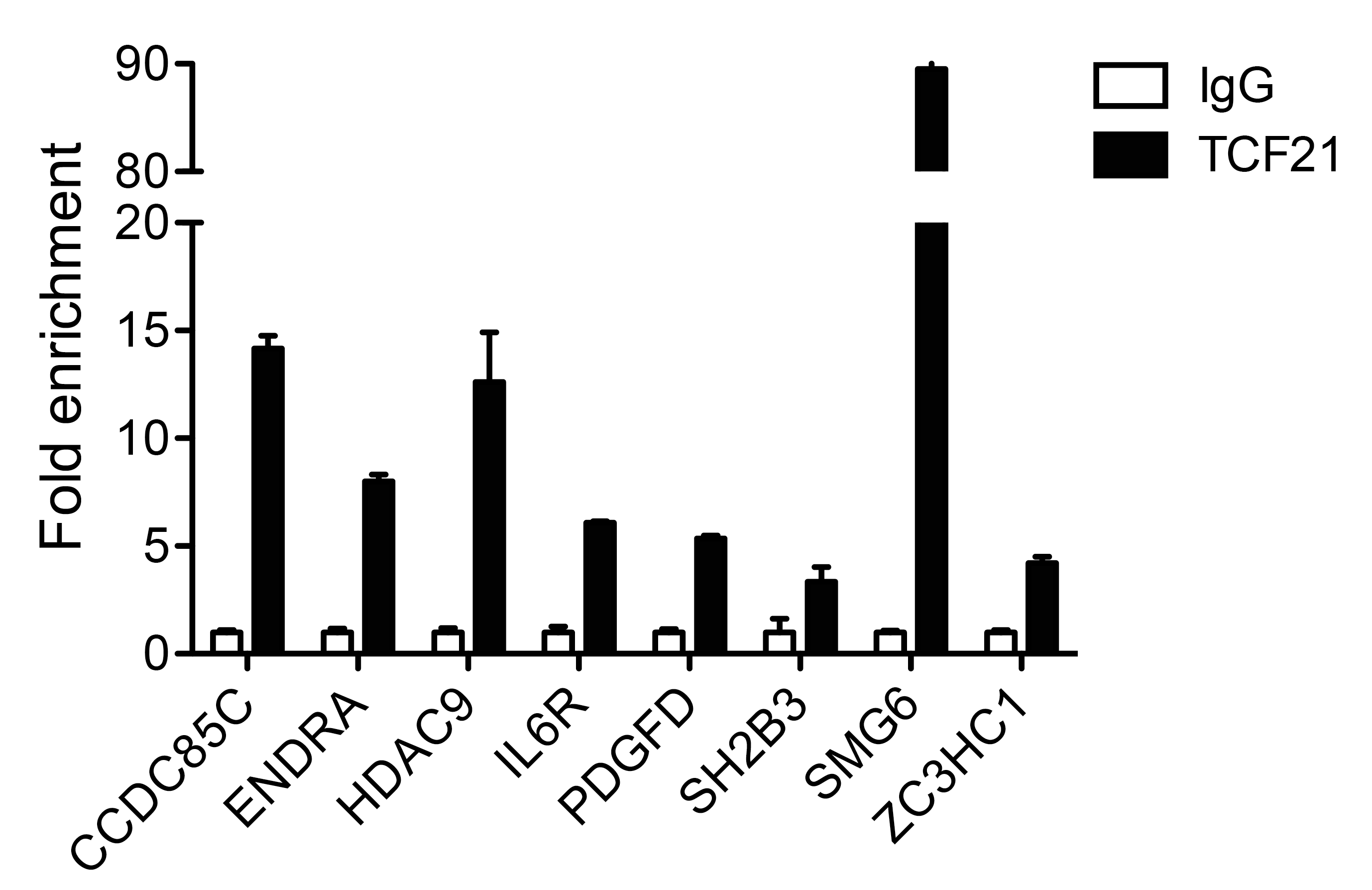

Supplement: S2 Fig — ChIP was performed with separately isolated chromatin from HCASMC derived from a different donor, employing PCR primers flanking a number of TCF21 peaks. (TIF) [file pgen.1005202.s002.tif]
