# Supplementary material for: Characterization of TCF21 Downstream Target Regions Identifies a Transcriptional Network Linking Multiple Independent Coronary Artery Disease Loci
Source: PLoS Genet. 2015 May 28;11(5):e1005202. doi: 10.1371/journal.pgen.1005202 (PMC4447360; doi:10.1371/journal.pgen.1005202)
Supplement: S1 Table — (PDF) [file pgen.1005202.s003.pdf]

Table S1. Representation of TCF21 ChIP-Seq peaks in regions of open chromatin.

Overlap between TCF21 ChIP-seq peaks and ATAC-Seq peaks					
TCF21 Ab_shared peaks	Overlapped peaks	No. ATAC-seq peaks (MACS fold-enrichment>200)	ATAC-seq background peaks (MACS fold-enrichment>50)	Enrichment P Value (Fisher's)	Fold Enrichment
3619*	2358	56193	159689	<1.0e-300	1.85

Overlap between TCF21 ChIP-seq peaks and HAoSMC DHS peaks

TCF21 Ab_shared peaks	Overlapped peaks	No. DHS peaks (P<0.05)	DHS background peaks	Enrichment P Value (Fisher's)	Fold Enrichment
4852*	4364	121731	580000	<1.0e-300	4.29

***Intersection of TCF21 Ab_Shared peaks and ATAC-Seq background peaks identified 3619 overlap regions, intersecton of TCF21 peaks with HAoSMC DNase hypersensitivity analyses identified 4852 overlap regions.**
