# Supplementary material for: Characterization of TCF21 Downstream Target Regions Identifies a Transcriptional Network Linking Multiple Independent Coronary Artery Disease Loci
Source: PLoS Genet. 2015 May 28;11(5):e1005202. doi: 10.1371/journal.pgen.1005202 (PMC4447360; doi:10.1371/journal.pgen.1005202)
Supplement: S2 Table — (PDF) [file pgen.1005202.s004.pdf]

Table S2. Enrichment of coexpression modules in TCF21 target genes.

Modules	Module Size	Overlapped_Count	P-value (Fisher's)	Fold Enrichment	Bonferroni Correction	Descriptions	Gene Ontology Terms (P<0.01, top 10 modules)
lightyellow	75	41	1.47E-17	3.938868313	3.98e-14*	'lightyellow:haec'	cytoskeletal adaptor activity
yellow	201	105	8.00E-39	3.763934955	2.16e-35*	'yellow:haec'	cytoskeletal protein binding
midnightblue	103	51	1.03E-18	3.56764653	2.80e-15*	'midnightblue:haec'	Human_Cancer_Microarray_OHS_802; cytoskeleton
green	185	85	1.09E-26	3.310518852	2.95e-23*	'green:haec'	extracellular space
4228	107	48	1.56E-15	3.232260298	4.22e-12*	'4228:liver'	extracellular matrix
grey60	75	31	1.10E-09	2.978168724	2.99e-06*	'grey60:haec'	MIF-mediated glucocorticoid regulation
darkorange	44	18	2.09E-06	2.94760101	0.00566*	'darkorange:haec'	positive regulation of transcription
4559	124	50	8.52E-14	2.905341497	2.30e-10*	'4559:adipose'	Human_Autoimmune_and_Inflamm_Response Gene_Array_HS_602.3
magenta	162	63	6.65E-16	2.802040466	1.80e-12*	'magenta:haec'	Cholera - Infection
brown	263	101	1.05E-23	2.767033986	2.84e-20*	'brown:haec'	vacuole

***Statistically significant.**
