# Supplementary material for: Characterization of TCF21 Downstream Target Regions Identifies a Transcriptional Network Linking Multiple Independent Coronary Artery Disease Loci
Source: PLoS Genet. 2015 May 28;11(5):e1005202. doi: 10.1371/journal.pgen.1005202 (PMC4447360; doi:10.1371/journal.pgen.1005202)
Supplement: S3 Table — (PDF) [file pgen.1005202.s005.pdf]

Table S3. Enrichment of candidate disease GWAS genes among the target genes of TCF21.

Methods	Target Genes	Overlapped GWAS genes	No. GWAS genes	No. total Genes	GWAS Genes Enrichment P Value (Fisher's)	Fold Enrichment
Ab1	12226	3422	8277	29775	0.27	1.01
Ab2	7150	2092	8277	29775	7.71e-4	1.05
Ab_Shared	7128	2092	8277	29775	3.98e-4	1.06
