# Supplementary material for: Characterization of TCF21 Downstream Target Regions Identifies a Transcriptional Network Linking Multiple Independent Coronary Artery Disease Loci
Source: PLoS Genet. 2015 May 28;11(5):e1005202. doi: 10.1371/journal.pgen.1005202 (PMC4447360; doi:10.1371/journal.pgen.1005202)
Supplement: S4 Table — (PDF) [file pgen.1005202.s006.pdf]

Table S4. Coordinates of TCF21 binding sites in CHD GWAS genes.

Gene Symbols	Gene Descriptions	TCF21 Peak counts	TCF21 Peaks (hg19)
AKAP13	A kinase (PRKA) anchor protein 13	1	chr15:86071120-86071439
ACAD10	acyl-Coenzyme A dehydrogenase family, member 10	1	chr12:112212000-112212402
ALDH2	aldehyde dehydrogenase 2 family (mitochondrial)	1	chr12:112212000-112212402
ALDH8A1	aldehyde dehydrogenase 8 family, member A1	1	chr6:135229940-135230900
APOA1	apolipoprotein A-I	1	chr11:116735252-116735667
APOA4	apolipoprotein A-IV	1	chr11:116735252-116735667
APOC3	apolipoprotein C-III	1	chr11:116735252-116735667
ATXN2	ataxin 2	1	chr12:111883850-111884393
AGBL1	ATP/GTP binding protein-like 1	1	chr15:87167781-87168252
BSND	Bartter syndrome, infantile, with sensorineural deafness (Barttin)	1	chr1:55438785-55439076
BNC2	basonuclin 2	1	chr9:16627919-16628365
BMP1	bone morphogenetic protein 1	1	chr8:22046008-22046557
CDH13	cadherin 13, H-cadherin (heart)	1	chr16:83563915-83564417
CELSR2	cadherin, EGF LAG seven-pass G-type receptor 2 (flamingo homolog, Drosophila)	1	chr1:109743759-109744199
CALCRL	calcitonin receptor-like	1	chr2:188229484-188229889
CACNA2D3	calcium channel, voltage-dependent, alpha 2/delta subunit 3	1	chr3:55034567-55035034
CCDC157	coiled-coil domain containing 157	2	chr22:30819839-30820350; chr22:30704470-30704871
COL4A1	collagen, type IV, alpha 1	1	chr13:110899908-110900338
COG5	component of oligomeric golgi complex 5	1	chr7:106820767-106821330
CDKN1A	cyclin-dependent kinase inhibitor 1A (p21, Cip1)	2	chr6:36595614-36596002; chr6:36646064-36646867
DNAH6	dynein, axonemal, heavy chain 6	1	chr2:85093262-85093611
EDNRA	endothelin receptor type A	2	chr4:148374703-148375073; chr4:148503674-148503981
FN1	fibronectin 1	2	chr2:216330884-216331237; chr2:216300051-216300397
FNDC1	fibronectin type III domain containing 1	1	chr6:159614914-159615400
FMN2	formin 2	1	chr1:240501166-240501507
GGCX	gamma-glutamyl carboxylase	4	chr2:85776860-85777313; chr2:85788397-85788736; chr2:85817281-85817736; chr2:85786834-85787154
GRIN3A	glutamate receptor, ionotropic, N-methyl-D-aspartate 3A	1	chr9:104478959-104479384
HEMGN	hemogen	1	chr9:100639022-100639369
HHIPL1	HHIP-like 1	1	chr14:100079253-100079689
HDAC9	histone deacetylase 9	2	chr7:18252385-18252802; chr7:18305269-18305550
INPP5D	inositol polyphosphate-5-phosphatase, 145kDa	1	chr2:233897585-233897974
IL6R	interleukin 6 receptor	2	chr1:154404053-154404466; chr1:154391982-154392306
JAZF1	JAZF zinc finger 1	2	chr7:27962837-27963274; chr7:27987337-27987676
KIAA1462	KIAA1462	2	chr10:30289388-30289999; chr10:30400591-30401022
KLF6	Kruppel-like factor 6	2	chr10:3826685-3827002; chr10:3819262-3819666
LIPA	lipase A, lysosomal acid, cholesterol esterase	1	chr10:91157771-91158063
LDLR	low density lipoprotein receptor	3	chr19:11275662-11276138; chr19:11253904-11254363; chr19:11236791-11237084
MTHFD1L	methylenetetrahydrofolate dehydrogenase (NADP+	1	chr6:151461847-151462324

	dependent) 1-like		
MAP4	microtubule-associated protein 4	2	chr3:48128562-48129233; chr3:48057208-48057624
MAP3K4	mitogen-activated protein kinase kinase kinase 4	2	chr6:161395112-161395914; chr6:161394378-161394798
MSI2	musashi homolog 2 (Drosophila)	2	chr17:55365457-55366031; chr17:55551139-55551468
MCL1	myeloid cell leukemia sequence 1 (BCL2-related)	1	chr1:150540020-150540455
NGF	nerve growth factor (beta polypeptide)	2	chr1:115873090-115873640; chr1:115825384-115825890
NRG1	neuregulin 1	2	chr8:32146023-32146545; chr8:32107856-32108223
ASIC2	acid-sensing (proton-gated) ion channel 2	1	chr17:31796834-31797322
HECTD4	HECT domain containing E3 ubiquitin protein l`igase 4	1	chr12:112559549-112559937
MIR4499	MIR4499	1	chr13:20993783-20994660
MIR548AV	MIR548AV	1	chr10:63752406-63752802
MIR548G	MIR548G	3	chr3:99764401-99765027; chr3:99620549-99620908; chr3:99751950-99752435
NME7	NME/NM23 family member 7	1	chr1:169336986-169337314
PRICKLE2-AS3	PRICKLE2 antisense RNA 3	2	chr3:64172488-64173194; chr3:64224689-64225145
WDR86-AS1	WDR86 antisense RNA 1	1	chr7:151063969-151064298
NUMB	numb homolog (Drosophila)	1	chr14:73702245-73702643
OBFC1	oligonucleotide/oligosaccharide-binding fold containing 1	2	chr10:105612476-105612876; chr10:105689126-105689608
OSM	oncostatin M	2	chr22:30704470-30704871; chr22:30634608-30635073
PCNXL3	pecanex-like 3 (Drosophila)	1	chr11:65420343-65420765
PHACTR1	phosphatase and actin regulator 1	1	chr6:13269345-13269882
PECAM1	platelet/endothelial cell adhesion molecule	1	chr17:62404480-62405115
PDGFRA	platelet-derived growth factor receptor, alpha polypeptide	7	chr4:54728318-54728852; chr4:54766070-54766499; chr4:54948320-54948707; chr4:54729334-54729668; chr4:54554263-54554772; chr4:55147082-55147583; chr4:54721257-54721585
PRICKLE2	prickle homolog 2 (Drosophila)	4	chr3:64172488-64173194; chr3:64059690-64060134; chr3:64224689-64225145; chr3:64252050-64252447
PROCR	protein C receptor, endothelial (EPCR)	1	chr20:33739605-33739987
PRKCA	protein kinase C, alpha	3	chr17:64455769-64456317; chr17:64417015-64417434; chr17:64463209-64463621
PPP2R3A	protein phosphatase 2 (formerly 2A), regulatory subunit B'', alpha	1	chr3:135685190-135685643
RAI1	retinoic acid induced 1	1	chr17:17725439-17725933
RND3	Rho family GTPase 3	1	chr2:151385819-151386168
ARHGAP26	Rho GTPase activating protein 26	3	chr5:142522263-142522599; chr5:142388401-142389134; chr5:142444833-142445160
SCARB1	scavenger receptor class B, member 1	2	chr12:125249282-125249782; chr12:125215096-125215522
SRR	serine racemase	1	chr17:2262955-2263333
SERPINH1	serpin peptidase inhibitor, clade H (heat shock protein 47), member 1, (collagen binding protein 1)	1	chr11:75265014-75265815
SARS	seryl-tRNA synthetase	1	chr1:109743759-109744199
SH2B3	SH2B adaptor protein 3	1	chr12:111883850-111884393
ADAMTS7	similar to hCG1991431; similar to COMPase; ADAM metallopeptidase with thrombospondin type 1 motif, 7	1	chr15:79052494-79052851
SMAD3	SMAD family member 3	3	chr15:67460532-67461098; chr15:67470896-67471589; chr15:67442610-67443257
SMG6	Smg-6 homolog, nonsense mediated mRNA decay factor (C. elegans)	5	chr17:2118754-2119591; chr17:2082955-2083474; chr17:2075422-2075856; chr17:2025895-2026247; chr17:2077661-2078596

SLC2A13	solute carrier family 2 (facilitated glucose transporter), member 13	1	chr12:40501166-40501786
SLC22A5	solute carrier family 22 (organic cation/carnitine transporter), member 5	3	chr5:131762056-131762559; chr5:131764632-131765122; chr5:131773162-131773775
SPC24	SPC24, NDC80 kinetochore complex component, homolog (S. cerevisiae)	3	chr19:11275662-11276138; chr19:11253904-11254363; chr19:11236791-11237084
SF3A1	splicing factor 3a, subunit 1, 120kDa	1	chr22:30704470-30704871
ST3GAL4	ST3 beta-galactoside alpha-2,3-sialyltransferase 4	3	chr11:126285013-126286074; chr11:126281343-126281773; chr11:126281946-126282328
ST8SIA1	ST8 alpha-N-acetyl-neuraminide alpha-2,8-sialyltransferase 1	1	chr12:22387586-22387923
SWAP70	SWAP switching B-cell complex 70kDa subunit	3	chr11:9781087-9781723; chr11:9786844-9787770; chr11:9809558-9810069
SMARCA4	SWI/SNF related, matrix associated, actin dependent regulator of chromatin, subfamily a, member 4	1	chr19:11026248-11026592
TOM1L2	target of myb1-like 2 (chicken)	2	chr17:17725439-17725933; chr17:17780195-17781588
TERT	telomerase reverse transcriptase	1	chr5:1204667-1205164
TNS1	tensin 1	2	chr2:218799210-218799667; chr2:218701018-218701871
TFPI	tissue factor pathway inhibitor (lipoprotein-associated coagulation inhibitor)	1	chr2:188412089-188412505
TRAFD1	TRAF-type zinc finger domain containing 1	1	chr12:112559549-112559937
VAMP8	vesicle-associated membrane protein 8 (endobrevin)	4	chr2:85776860-85777313; chr2:85788397-85788736; chr2:85817281-85817736; chr2:85786834-85787154
SKI	v-ski sarcoma viral oncogene homolog (avian)	2	chr1:2160492-2160846; chr1:2139706-2140191
ZFPM2	zinc finger protein, multitype 2	1	chr8:106285901-106286274
ZC3HC1	zinc finger, C3HC-type containing 1	1	chr7:129687632-129687947
