# Supplementary material for: Characterization of TCF21 Downstream Target Regions Identifies a Transcriptional Network Linking Multiple Independent Coronary Artery Disease Loci
Source: PLoS Genet. 2015 May 28;11(5):e1005202. doi: 10.1371/journal.pgen.1005202 (PMC4447360; doi:10.1371/journal.pgen.1005202)
Supplement: S5 Table — (PDF) [file pgen.1005202.s007.pdf]

Table S5. GO term enrichment for CAD genes associated with TCF21 peaks.

Term	P-Value	Fold Enrichment
GO:0030335~positive regulation of cell migration	4.53e-06	15.88059701
GO:0040017~positive regulation of locomotion	7.92e-06	14.42217484
GO:0051272~positive regulation of cell motion	7.92e-06	14.42217484
GO:0030334~regulation of cell migration	1.76e-05	9.557891018
GO:0040012~regulation of locomotion	3.99e-05	8.412935323
GO:0051270~regulation of cell motion	4.13e-05	8.369344985
GO:0051174~regulation of phosphorus metabolic process	1.11e-04	4.579412217
GO:0019220~regulation of phosphate metabolic process	1.11e-04	4.579412217
GO:0001932~regulation of protein amino acid phosphorylation	1.93e-04	8.169786904
GO:0008283~cell proliferation	2.51e-04	4.630973573
GO:0042325~regulation of phosphorylation	4.09e-04	4.332842227
GO:0008202~steroid metabolic process	4.44e-04	6.996896705
GO:0032268~regulation of cellular protein metabolic process	4.63e-04	4.259714088
GO:0010033~response to organic substance	6.69e-04	3.360506759
GO:0016125~sterol metabolic process	0.0014796	9.995566721
GO:0015697~quaternary ammonium group transport	0.001499223	50.47761194
GO:0030198~extracellular matrix organization	0.001648214	9.707233065
GO:0042445~hormone metabolic process	0.001767778	9.524077725
GO:0002687~positive regulation of leukocyte migration	0.002362711	40.38208955
GO:0034381~lipoprotein particle clearance	0.002691762	37.85820896
GO:0031399~regulation of protein modification process	0.003100631	4.791095371
GO:0032103~positive regulation of response to external stimulus	0.0037375	12.61940299
GO:0009611~response to wounding	0.004099491	3.428667981
GO:0002685~regulation of leukocyte migration	0.004208717	30.28656716
GO:0035295~tube development	0.004344179	5.506648575
GO:0032270~positive regulation of cellular protein metabolic process	0.005529646	5.199410672
GO:0015695~organic cation transport	0.005551715	26.33614536
GO:0032496~response to lipopolysaccharide	0.006273626	10.48885443
GO:0010817~regulation of hormone levels	0.00629046	6.685776416
GO:0051247~positive regulation of protein metabolic process	0.006585537	4.985443155
GO:0043062~extracellular structure organization	0.008202985	6.193572017
GO:0006694~steroid biosynthetic process	0.00824032	9.50166813
GO:0002237~response to molecule of bacterial origin	0.008508786	9.391183617
GO:0001934~positive regulation of protein amino acid phosphorylation	0.009344889	9.074626866
GO:0070508~cholesterol import	0.009734096	201.9104478
GO:0008203~cholesterol metabolic process	0.010227463	8.77871512
GO:0042592~homeostatic process	0.010324388	2.688554564
GO:0016477~cell migration	0.011069022	4.38935756
GO:0042327~positive regulation of phosphorylation	0.01180299	8.326204031
GO:0010562~positive regulation of phosphorus metabolic process	0.012811735	8.07641791
