# Supplementary material for: Characterization of TCF21 Downstream Target Regions Identifies a Transcriptional Network Linking Multiple Independent Coronary Artery Disease Loci
Source: PLoS Genet. 2015 May 28;11(5):e1005202. doi: 10.1371/journal.pgen.1005202 (PMC4447360; doi:10.1371/journal.pgen.1005202)
Supplement: S6 Table — (PDF) [file pgen.1005202.s008.pdf]

Table S6. GO term enrichment for all CAD genes.

Term	P-Value	Fold Enrichment
GO:0055088~lipid homeostasis	1.26e-11	25.15348208
GO:0015918~sterol transport	1.72e-11	31.51910531
GO:0030301~cholesterol transport	1.72e-11	31.51910531
GO:0055092~sterol homeostasis	8.43e-10	27.62068966
GO:0042632~cholesterol homeostasis	8.43e-10	27.62068966
GO:0034381~lipoprotein particle clearance	2.47e-09	51.02155172
GO:0033344~cholesterol efflux	8.20e-09	42.96551724
GO:0006869~lipid transport	4.02e-08	9.65136742
GO:0010876~lipid localization	9.13e-08	8.913683286
GO:0051241~negative regulation of multicellular organismal process	1.43e-07	8.533221194
GO:0016125~sterol metabolic process	1.84e-07	11.54660294
GO:0034367~macromolecular complex remodeling	5.70e-07	34.9862069
GO:0034369~plasma lipoprotein particle remodeling	5.70e-07	34.9862069
GO:0034368~protein-lipid complex remodeling	5.70e-07	34.9862069
GO:0032371~regulation of sterol transport	5.70e-07	34.9862069
GO:0032374~regulation of cholesterol transport	5.70e-07	34.9862069
GO:0008203~cholesterol metabolic process	1.10e-06	11.40854573
GO:0008202~steroid metabolic process	1.15e-06	6.927961762
GO:0006641~triglyceride metabolic process	1.56e-06	18.98476343
GO:0030334~regulation of cell migration	1.71e-06	7.590695776
GO:0006639~acylglycerol metabolic process	3.44e-06	16.66009852
GO:0006638~neutral lipid metabolic process	3.88e-06	16.32689655
GO:0006662~glycerol ether metabolic process	4.36e-06	16.00676133
GO:0032368~regulation of lipid transport	4.89e-06	23.32413793
GO:0040012~regulation of locomotion	5.38e-06	6.681393678
GO:0018904~organic ether metabolic process	5.49e-06	15.4027326
GO:0051270~regulation of cell motion	5.63e-06	6.646775058
GO:0043691~reverse cholesterol transport	8.34e-06	36.44396552
GO:0030335~positive regulation of cell migration	1.04e-05	10.48275862
GO:0034384~high-density lipoprotein particle clearance	1.17e-05	77.74712644
GO:0042592~homeostatic process	1.74e-05	3.105744065
GO:0040017~positive regulation of locomotion	1.95e-05	9.520056298
GO:0051272~positive regulation of cell motion	1.95e-05	9.520056298
GO:0032372~negative regulation of sterol transport	2.04e-05	66.64039409
GO:0032375~negative regulation of cholesterol transport	2.04e-05	66.64039409
GO:0070328~triglyceride homeostasis	3.25e-05	58.31034483
GO:0010033~response to organic substance	3.55e-05	3.073222057
GO:0010884~positive regulation of lipid storage	4.84e-05	51.83141762
GO:0019220~regulation of phosphate metabolic process	6.20e-05	3.606825453
GO:0051174~regulation of phosphorus metabolic process	6.20e-05	3.606825453
