# Supplementary material for: Characterization of TCF21 Downstream Target Regions Identifies a Transcriptional Network Linking Multiple Independent Coronary Artery Disease Loci
Source: PLoS Genet. 2015 May 28;11(5):e1005202. doi: 10.1371/journal.pgen.1005202 (PMC4447360; doi:10.1371/journal.pgen.1005202)
Supplement: S7 Table — (PDF) [file pgen.1005202.s009.pdf]

**Table S7. GO term enrichment for TCF21 CAD genes analyzed with background of all CAD genes.**

| Term | P-Value | Fold Enrichment |
|---|---|---|
| GO:0008270~zinc ion binding | 0.002598762 | 2.549350649 |
| GO:0030029~actin filament-based process | 0.014987891 | 13.50746269 |
| GO:0030036~actin cytoskeleton organization | 0.014987891 | 13.50746269 |
| GO:0002684~positive regulation of immune system process | 0.030705039 | 5.402985075 |
| GO:0010604~positive regulation of macromolecule metabolic process | 0.031088666 | 2.513016314 |
| GO:0008134~transcription factor binding | 0.034388955 | 3.813131313 |
| GO:0007242~intracellular signaling cascade | 0.039529561 | 2.078071183 |
| GO:0048584~positive regulation of response to stimulus | 0.040054148 | 4.911804613 |
| GO:0051098~regulation of binding | 0.045386544 | 8.104477612 |
| GO:0051101~regulation of DNA binding | 0.045386544 | 8.104477612 |
| GO:0006954~inflammatory response | 0.050675601 | 4.502487562 |
| GO:0051240~positive regulation of multicellular organismal process | 0.050675601 | 4.502487562 |
